# A Systematic Review of Instruments to Assess Organizational Readiness for Knowledge Translation in Health Care

**DOI:** 10.1371/journal.pone.0114338

**Published:** 2014-12-04

**Authors:** Marie-Pierre Gagnon, Randa Attieh, El Kebir Ghandour, France Légaré, Mathieu Ouimet, Carole A. Estabrooks, Jeremy Grimshaw

**Affiliations:** 1 Research Center of the Centre Hospitalier Universitaire de Québec, Québec, Canada; 2 Faculty of Nursing, Université Laval, Québec, Canada; 3 Department of Family Medicine, Université Laval, Québec, Canada; 4 Department of Political Science, Université Laval, Québec, Canada; 5 Faculty of Nursing, University of Alberta, Edmonton, Canada; 6 Ottawa Hospital Research Institute, Ottawa, Canada; 7 Faculty of Medicine, University of Ottawa, Ottawa, Canada; National University of Singapore, Singapore

## Abstract

**Background:**

The translation of research into practices has been incomplete. Organizational readiness for change (ORC) is a potential facilitator of effective knowledge translation (KT). However we know little about the best way to assess ORC. Therefore, we sought to systematically review ORC measurement instruments.

**Methods:**

We searched for published studies in bibliographic databases (Pubmed, Embase, CINAHL, PsychINFO, Web of Science, etc.) up to November 1^st^, 2012. We included publications that developed ORC measures and/or empirically assessed ORC using an instrument at the organizational level in the health care context. We excluded articles if they did not refer specifically to ORC, did not concern the health care domain or were limited to individual-level change readiness. We focused on identifying the psychometric properties of instruments that were developed to assess readiness in an organization prior to implementing KT interventions in health care. We used the Standards for Educational and Psychological Testing to assess the psychometric properties of identified ORC measurement instruments.

**Findings:**

We found 26 eligible instruments described in 39 publications. According to the Standards for Educational and Psychological Testing, 18 (69%) of a total of 26 measurement instruments presented both validity and reliability criteria. The *Texas Christian University –ORC (TCU-ORC) scale* reported the highest instrument validity with a score of 4 out of 4. Only one instrument, namely the *Modified Texas Christian University – Director version (TCU-ORC-D)*, reported a reliability score of 2 out of 3. No information was provided regarding the reliability and validity of five (19%) instruments.

**Conclusion:**

Our findings indicate that there are few valid and reliable ORC measurement instruments that could be applied to KT in the health care sector. The TCU-ORC instrument presents the best evidence in terms of validity testing. Future studies using this instrument could provide more knowledge on its relevance to diverse clinical contexts.

## Background

Health care systems are constantly changing, sometimes in subtle ways but at other times in major or even disruptive ways, in response to new public health policy, emerging market necessities, and technological advances [1]. At the same time, there is increasing international interest in organizational change as a lever for health care improvement [2]. Generally, organizational change is defined as any modification in organizational composition, structure, or behavior [3]. With the persistent gaps between research and practice in health care systems, knowledge translation (KT) has gained significance and importance in answering these challenges [4]. KT is defined as the methods for closing the knowledge-to-action gaps [5]. While organizational characteristics have been shown to influence research utilization in practice, organizations aiming to improve care require an adequate level of organizational readiness in order to implement research-based knowledge [6], [7].

According to Weiner *et al*. [8], Organizational Readiness for Change (ORC) is defined as a key overarching concept to assess organizational members’ collective motivation and capability to implement change. Readiness for change is a comprehensive attitude influenced simultaneously by the nature of the change, the change process, the organization’s context and the attributes of individuals [9]. “Readiness” is considered a multilevel latent construct [10]. It is thus possible to capture the concept of readiness by breaking it down into its measurable core concepts. Change management researchers have emphasized the importance of establishing ORC and recommended various ways to prepare for change [10], [11]. As stated by Armenakis and Harris [12], when ORC is high, organizational members invest more in the change effort and exhibit greater persistence to overcome obstacles and setbacks.

The translation of research into practices has been incomplete [2]. ORC is a potential facilitator of effective KT. As highlighted by Wise et al. [13], with the lack of understanding of organizational and/or system readiness for implementing change and knowledge of effective strategies to increase readiness, there is a potential that change implementation be unsuccessful. As stated by Greenhalgh [14], system readiness for implementing change refers to when organizations move toward a state of readiness to assess and anticipate the impact of a change. Organizational readiness has emerged as a key concept in the KT literature. For instance, the development and evaluation of implementation strategies for primary prevention programs and policies addressing chronic disease would benefit from the assessment of organizational readiness in the process of integrating knowledge about the practice setting [15].

According to Kotter [16], half the failures to implement organizational change occur because organizational leaders failed to establish the level of readiness. An organization may be amenable to change in general but not ready or willing to assimilate a particular change [14]. In their extensive review, Weiner et al. [8] examined how ORC has been defined as a critical precursor to the successful implementation of complex changes in health care settings and how it has been measured in health services and in other fields. Holt et al [9] and Weiner et al [8] have summarized existing instruments and methods to measure readiness for change in health services and other fields [8], [9]. Focusing on the instrument’s content and psychometric properties, these reviews brought up the limited evidence of reliability and validity of most currently available ORC measurement instruments [8], [9]. However, the choice of an instrument in many cases is not a simple matter of selecting the most valid one. Available valid measurement instruments often examined readiness narrowly, omitting one or more conceptual issues that are important parts of a comprehensive evaluation of readiness [17]. Also we know little about instruments specifically designed to assess organizational readiness for KT, defined as a healthcare organization’s potential for implementing evidence-based practices, and it is not clear whether existing instruments could be useful to support health care decision makers in their implementation of evidence-based interventions in real life settings [18]. To achieve these goals, we sought to review ORC measurement instruments that could apply to knowledge translation in health care.

## Methods

### Data sources and search strategy

We conducted a mixed method systematic review of the literature on ORC measurement instruments in health care [19]. We followed the PRISMA checklist [20]. An information specialist developed the search strategy on PubMed and then translated it across the other databases. The search strategy included four categories of keywords: (i) Readiness, (ii) Commitment and Change, (iii) Organization and Administration and (iv) Health and Social Services (Table 1). We searched the following databases: Pubmed, Embase, CINAHL, PsychINFO, Web of Sciences (SCI and SSCI), Business Source Premier, ABI/Inform, and Sociological Abstracts.

**Table 1:** Search strategy.

**Pubmed**
1- Readiness: Readiness[TIAB]
2- Commitment AND Change: (Commitment[TIAB] OR Preparedness[TIAB] OR Acceptance[TIAB] OR Willingness[TIAB]) AND (Change[TI] OR Changing[TI] OR Organizational Innovation[MH:NOEXP] OR Organizational Innovation*[TIAB] OR Organisational Innovation*[TIAB] OR Organizational change*[TIAB] OR Organisational change*[TIAB] OR Institutional change*[TIAB] OR Institutional innovation*[TIAB]) OR “Stages of change” [TIAB]
3- Organization and Administration: “Organization and Administration:” [SH:NOEXP] OR Organizational Innovation[MH:NOEXP] OR Organisation*[TIAB] OR Organization*[TIAB] OR Institutional*[TIAB]
4- Health and social services: N/A
5- (#1 AND #2) AND #3
**Embase**
1- Readiness: Readiness: ti,ab
2- Commitment AND Change: ((commitment: ab,ti OR preparedness: ab,ti OR acceptance: ab,ti OR willingness: ab,ti) AND (change: ti OR changing: ti OR ‘organizational innovation’: ab,ti OR ‘organizational innovations’: ab,ti OR ‘organisational innovation’: ab,ti OR ‘organisational innovations’: ab,ti OR ‘organizational change’: ab,ti OR ‘organizational changes’: ab,ti OR ‘organisational change’: ab,ti OR ‘organisational changes’: ab,ti OR ‘institutional change’: ab,ti OR ‘institutional changes’: ab,ti OR ‘institutional innovation’: ab,ti OR ‘institutional innovations’: ab,ti)) OR ‘stages of change’: ab,ti
3- Organization and Administration: ‘organization’/exp OR organisation*: ab,ti OR organization*: ab,ti OR institutional*: ab,ti
4- Health and social services: N/A
5- (#1 AND #2) AND #3 Limited to Embase
**CINAHL (Ebsco)**
1- Readiness: TI Readiness OR AB Readiness
2- Commitment AND Change: (TI (Commitment OR Preparedness OR Acceptance OR Willingness) OR AB (Commitment OR Preparedness OR Acceptance OR Willingness)) AND (TI Change OR TI Changing OR TI (Organizational Innovation* OR Organisational Innovation* OR Organizational change* OR Organisational change* OR Institutional change* OR Institutional innovation*) OR AB (Organizational Innovation* OR Organisational Innovation* OR Organizational change* OR Organisational change* OR Institutional change* OR Institutional innovation*) OR MH Organizational Change) OR TI “Stages of change” OR AB “Stages of change”
3- Organization and Administration: MH Organizational Change OR MH Organizations+ OR AB Organisation* OR TI Organisation* OR AB Organization* OR TI Organization* OR AB Institutional* OR TI Institutional*
4- Health and social services: N/A
5- (#1 AND #2) AND #3 Limited to “Peer Reviewed” Exclude Medline records
**PsycINFO**
1- Readiness: ti = readiness or ab = readiness
2- Commitment AND Change: ((ti = (Commitment OR Preparedness OR Acceptance OR Willingness) OR ab = (Commitment OR Preparedness OR Acceptance OR Willingness)) AND (ti = Change OR ti = Changing OR ti = (“Organizational Innovation” OR “Organisational Innovation” OR “Organizational change” OR “Organisational change” OR “Institutional change” OR “Institutional innovation”) OR ab = (“Organizational Innovation” OR “Organisational Innovation” OR “Organizational change” OR “Organisational change” OR “Institutional change” OR “Institutional innovation”) OR it = “Organizational Change”)) OR ti = “Stages of change” OR ab = “Stages of change”
3- Organization and Administration: it = “Organizational Change” OR it = Organizations OR ti = (Organization* OR Organisation* OR Institutional*) OR ab = (Organization* OR Organisation* OR Institutional*)
4- Health and social services: N/A
5- (#1 AND #2) AND #3 Limited Peer-Reviewed Journals only
**Web of science (SCI and SSCI)**
1- Readiness: TS = Readiness
2- Commitment AND Change: TS = (Commitment OR Preparedness OR Acceptance OR Willingness) AND (TI = (Change OR Changing) OR TS = ((“Organizational Innovation*”) OR (“Organisational Innovation*”) OR (“Organizational change*”) OR (“Organisational change*”) OR (“Institutional change*”) OR (“Institutional innovation*”))) OR TS = (“Stages of change”)
3- Organization and Administration: TS = (Organization* OR Organisation* OR Institutional*)
4- Health and social services: TS = (Health* OR Medic* OR (“Social service”))
5- (#1 AND #2) AND #3 AND 4
**Business Source Premier (EBSCO)**
1- Readiness: TI Readiness OR AB Readiness
2- Commitment AND Change: (TI (Commitment OR Preparedness OR Acceptance OR Willingness) OR AB (Commitment OR Preparedness OR Acceptance OR Willingness)) AND (TI Change OR TI Changing OR TI (Organizational Innovation* OR Organisational Innovation* OR Organizational change* OR Organisational change* OR Institutional change* OR Institutional innovation*) OR AB (Organizational Innovation* OR Organisational Innovation* OR Organizational change* OR Organisational change* OR Institutional change* OR Institutional innovation*) OR DE “Organizational Change”) OR TI “Stages of change” OR AB “Stages of change”
3- Organization and Administration: DE “ORGANIZATION” or DE “ORGANIZATIONAL change” OR AB (Organisation* OR Organization* OR Institutional*) OR TI (Organisation* OR Organization* OR Institutional*)
4- Health services and social: SU Health* OR TI Health* OR AB Health* OR SU Medic* OR TI Medic* OR AB Medic* OR DE “Social service” OR TI Social service* OR AB Social service*
5- (#1 AND #2) AND #3 AND 4 Limited to Scholarly (Peer Reviewed) Journals
**Proquest ABI/Inform**
1- Readiness: TI(Readiness) OR AB(Readiness)
2- Commitment AND Change: TI(Commitment OR Preparedness OR Acceptance OR Willingness) OR AB(Commitment OR Preparedness OR Acceptance OR Willingness) AND (TI (Change OR Changing OR “Organizational Innovation*” OR “Organisational Innovation*” OR “Organizational change*” OR “Organisational change*” OR “Institutional change*” OR “Institutional innovation*”) OR AB(“Organizational Innovation*” OR “Organisational Innovation*” OR “Organizational change*” OR “Organisational change*” OR “Institutional change*” OR “Institutional innovation*”) OR SU(“Organizational change”)) OR TI “Stages of change” OR AB “Stages of change”
3- Organization and Administration: SU(“Organizational change”) OR SU(Organization) OR TI(Organisation* OR Organization* OR Institutional*) OR AB(Organisation* OR Organization* OR Institutional*)
4- Health and social services: SU(Health*) OR TI(Health*) OR AB(Health*) OR SU(Medic*) OR TI(Medic*) OR AB(Medic*) OR SU(Social services) OR TI(Social services) OR AB(Social services)
5- (#1 AND #2) AND #3 AND 4 Limited to “Peer Reviewed”
**Sociological Abstracts database**
1- Readiness: KW = Readiness
2- Commitment AND Change: KW = (Commitment OR Preparedness OR Acceptance OR Willingness) AND (TI = (Change OR Changing) OR KW = ((“Organizational Innovation*”) OR (“Organisational Innovation*”) OR (“Organizational change*”) OR (“Organisational change*”) OR (“Institutional change*”) OR (“Institutional innovation*”))) OR KW = (“Stages of change”)
3- Organization and Administration: KW = (Organization* OR Organisation* OR Institutional*)
4- Health and social services: KW = (Health* OR Medic* OR (“Social service”))
5- (#1 AND #2) AND #3 AND 4 Limited to “Peer Reviewed”

### Screening and eligibility criteria

Pairs of authors (RA, EKG, MPG) independently screened the published literature by reviewing their titles and abstracts. Then, two authors (RA, EKG) appraised the full text of each study independently. We also planned resolving discrepancies between authors through discussion, or involving a third reviewer as arbiter, if necessary. We retained articles published in all languages, as long as they had an abstract in English, Finnish, French, Portuguese, Spanish or Swedish (languages that team members speak). We limited our search to articles published before November 1^st^, 2012, which explicitly referred to the health care domain and applied the concept of ORC or its related terms (preparedness, commitment, or willingness to change). We included articles that developed ORC measures and/or empirically assessed ORC. It was an imperative that selected instruments should be based on conceptual models/frameworks of ORC relevant to KT in healthcare sector at the organizational level, as provided in our systematic review of theoretical components of ORC [21]. We excluded articles if they did not refer specifically to organizational readiness or any of its related concepts, did not concern the health care domain, were limited to individual-level measure of readiness, or were in languages other than the ones identified above. Finally, a third reviewer (MPG) checked all the excluded and included studies.

### Data extraction

We first compiled the descriptive (e.g., author, year, type of study, setting, underlying model/theory and level of analysis) (Table 2) and the psychometric (e.g., source of instrument, constructs/items, validity and reliability) properties of organizational readiness instruments (Table 3). We then appraised the extent to which evidence exists for each identified instruments’ reliability and validity with a checklist that we developed based on the Standards for Educational and Psychological Testing (SEPT) published in 1999 by the American Educational Research Association (AERA), the American Psychological Association (APA) and the National Council on Measurement in Education (NCME) [22]. A main reason for choosing the SEPT as a guiding framework was because it provides a contemporary conceptualization of validity and reliability [23].

**Table 2:** Descriptive Characteristics of Organizational Readiness Measurement Instruments.

Instrument	Authors	Year	Type of study	Setting	Underlying theory/model	Level of analysis
1- a Organizational readiness for change scale (ORC) [25]	Lehman et al.	2002	Empirical	Clinical center (CTN), Drug treatment program	Program Change Model (TCU-PCM)	Organizational, Individual
1-b Extended Organizational readiness for change scale (ORC) [25], [30]	Lehman et al.	2005	Methodological	Alcohol and Other Drug Abuse Services, Mental health services	Program change model (TCU-PCM)	Organizational, Individual
1-c Modified ORC scale [50]	Barwick et al.	2005	Empirical	Mental health Organizations	Not specified	Organizational
1-d TCU-ORC scale [25]	Lehman, Greener & Simpson	2002	Methodological	Addiction Technology Transfer Centers (and several other drug treatment programs)	Revised TCU-PCM	Organizational Individual
1-e Modified Texas Christian University – Director version (TCU-ORC-D) [29]	Chabot et al.	2008	Methodological	Local health organizations	ORC conceptual framework	Organizational Individual
1-f Functional Organizational Readiness For Change Evaluation (FORCE) [51]	Devereaux et al.	2006	Empirical	Hospitals	No	Organizational
2- The Medical Organizational Readiness For Change (MORC) [52]	Bohman et al.	2002	Empirical	Trauma center (Community health program + Emergency center)	TCU-PCM	Organizational Individual
3- Organizational readiness to change assessment instrument (ORCA) [53]	Helfrich et al.	2009	Methodological	VA medical centers	Promoting Action on Research in Health Services (PARiHS)	Organizational
4- The organizational change questionnaire [26]	Bouckenooghe et al.	2009	Methodological	Organizations (healthcare, medical services)	Human relations perspective	Organizational Individual
5- Organizational Information Technology Innovation Readiness Scale (OITIRS) [35]	Snyder-Halpern	1996	Methodological	Healthcare (community hospitals)	Organizational Information Technology/System Innovation Model (OITIM)	Organizational
6- Perceived organizational readiness for change (PORC) [36]	Armenakis, Harris and Mossholder	1993	Empirical	Public sector organizations	The concept of perceived ORC	Organizational
7- Proactive Organizational Change: Assessing Critical Success Factors [37]	Nelson et al.	1999	Empirical	Public health agencies	No	Organizational
8- Organizational Telehealth readiness assessment tool [38]	Jennett et al.	2004	Methodological	Rehabilitation sectors	Readiness model	Organizational Individual
9- e-Health Readiness measure [39]	Poissant, Touré & Swaine	2007	Methodological	Rehabilitation Centre (CRLB)	No	OrganizationalIndividual
10- Organization Culture and Readiness Survey (OCRS) [40]	Melnyk et al.	2008	Methodological	Faith-based hospital was located in a moderate sized city	Advancing research and clinical practice through close collaboration (ARCC)	Organizational Individual
11- Team Climate Inventory (TCI) [27]	Anderson &West	1994	Empirical	NHS trusts	No	Group level
12- Sociotechnical System Assessment Surveys (STSAS) [41]	Pasmore	1988	Empirical	Tertiary care hospitals	Sociotechnical system theory	Organizational
13- Computerized Physician Order Entry (CPOE) [42]	Stablein et al.	2001	Empirical	Hospitals	No	Organizational
14- Safer patients initiatives (SPI) [43]	Burnett et al.	2010	Empirical	NHS organizations	No	Organizational
15- Not specified [44]	Demiris et al.	2007	Empirical	Hospitals	No	Organizational
16- Not specified [45]	Hamilton et al.	2010	Empirical	VA medical centers	No	Organizational
17- Psychometrically sound survey instrument [28]	Holt, Armenakis, Feild & Harris	2007	Methodological	Public & private sectors	Comprehensive Measurement Model (CMM	Organizational Individual
18- Not specified [34]	Kristensen & Nohr	2000	Methodological	Healtcare org (Surgical gastroenterology department	Lorenzie’s	Organizational Individual Group
19- Geriatric Institutional Assessment Profile (GIAP) [46], [47]	Boltz et al.	2002	Methodological	Organization (Hospitals)	No	Organizational Individual
20- Long-Term care (LTC) readiness tool [48]	Cherry	2011	Methodological	Organization (long term care facilities)	Not specified	Organizational Individual
21- Not specified [49]	Bobiak et al.	2009	Empirical	Organizations (primary care settings)	Practice Change Model (PCM)	Organizational

**Table 3:** Psychometric Properties of Organizational Readiness Measurement Instruments.

Instrument’sname	Source ofthe instrument	Constructs	Number ofItems	Validity				Reliability			
				Content	Criterion	Construct	Face	Internal consistency	Parallel forms	Test-Retest	Split half
1-a Organizationalreadiness forchange scale(ORC)[25], [54], [55]	Lehmanet al (2002)	1) Motivation forchange “motivationalforces influencingthe need for change”	3 items	None	Predictive	None	None	Cronbach alpha (0.69–0.88)	None	None	None
		2) Institutional resources“adequacy of resourcesneeded for daily activitiesand forsupporting change”	5 items								
		3) Staff attributes“efficacy and adaptabilityof staff and leaders”	4 items								
		4) Organizational climate“an environment thatencourages adoption ofpractices to make changessustainable”	6 items								
1-b ExtendedOrganizationalreadiness forchange scale(ORC) [25], [30]	Lehmanet al (2002)	1) Motivation for change“motivational forcesinfluencing the need forchange”	3 items	None	Concurrent	Convergent	None	Cronbach alpha (0.69–0.88)	None	None	None
		2) Institutional resources“adequacy of resourcesneeded for daily activitiesand for supporting change”	5 items								
		3) Staff attributes“efficacy and adaptabilityof staff and leaders”	4 items								
		4) Organizational climate“an environment thatencourages adoption ofpractices to make changessustainable”	6 items								
		5) Trainingexposure & utilization	2 items								
1-c ModifiedORC scale [50]	Barwicket al (2005)	1) Motivation and readinessfor change “motivationalforces influencing the needfor change”	3 items	None	None	None	None	Cronbach alpha(0.60)	None	None	None
		2) Institutional resources“adequacy of resourcesneeded for daily activitiesand for supporting change”	5 items								
		3) Personality attributesof the staff “efficacy andadaptability of staff andleaders”	4 items								
		4) Organizational climate“an environment thatencourages adoption ofpractices to make changessustainable”	6 items								
1-d TCU-ORCscale [25]	Lehmanet al (2002)	1) Motivation for change“motivational forcesinfluencing the need forchange”	3 items (α = 0.69)	Expert judges	None	EFA	Yes	Cronbach alpha(0.69–0.88)	None	None	None
		2) Institutional resources“adequacy of resourcesneeded for daily activitiesand for supporting change”	5 items (α = 0.71)								
		3) Staff attributes“efficacy and adaptabilityof staff and leaders”	4 items(α = 0.70)								
		4) Organizational climate“an environment thatencourages adoption ofpractices to make changessustainable”	6 items (α = 0.88)								
1-e ModifiedTexas ChristianUniversity –Directorversion(TCU-ORC-D)[29]	Chabotet al (2008)	1) Motivations “refer to theforces influencing theadoption of a specificchange”	3 items	5 Expert judges	None	None	Yes	Cronbach alpha(0.41–0.95)	None	Yes	None
		2) Resources “such asinformation technology(IT) are known to have apositive influence on theadoption of an organizationalinnovation”	4 items								
		3) Leaders’ attributes“influence workers’motivation andorganizational climate”	4 items								
		4) Organizational Climate“refers to members’ sharedperceptions (aggregatedratings) of the organizationalenvironment”	8 items								
1-f FunctionalOrganizationalReadiness ForChangeEvaluation(FORCE) [51]	Devereauxet al. (2006)	1) Motivation for change“motivational forcesinfluencing the need forchange”	3 items	None	None	None	Yes	Cronbach alpha(>0.70)	None	None	None
		2) Access to resources“adequacy of resources”	5 items								
		3) Staff attributes “efficacy and adaptability of staff and leaders”	4 items								
		4) Organizational climate“an environment thatencourages adoption ofpractices to make changessustainable”	6 items								
		5) Trainingexposure/utilization“convenience of trainingopportunities and the use ofknowledge and skillsacquired as part of thetraining”	4 items								
2- The MedicalOrganizationalReadiness ForChange(MORC) [52]	Bohmanet al. (2002)	1) Need for ExternalGuidance “Activities inwhich organization needsadditional guidance”	5 items	None	None	None	None	Cronbach alpha(0.67–0.96)	None	None	None
		2) Pressure to Change “staffmembers’ perceptions of whoseeks changes in theorganization”	8 items								
		3) Organizational Readinessto Change “Organization’swillingness and ability toincorporate changesnecessary for technologytransfer”	6 items								
		4) Individual Readiness toChange “Individual staffmembers’ ability toincorporate change basedon their perception”	7 items								
		5) Workgroup Functioning“staff members’ ability toincorporate change based ontheir ability to work together”	5 items								
		6) Work Environment“Perceived amount andflexibility of rules and workoverload”	6 items								
		7) Autonomy Support “Levelof respect for and support forindividual staff members’knowledge, ability, andprofessional judgment”	5 items								
		8) Alcohol and Drug Focus“recognition of alcohol anddrug issues related toindividual training and workenvironment”	3 items								
3- Organizationalreadiness tochangeassessmentinstrument(ORCA) [53]	Helfrichet al (2009)	1) Evidence “ the strengthand nature of the evidenceas perceived by multiplestakeholders”	4 items (α = 0.74)	None	None	ConvergentEFACFA	None	Cronbach alpha (0.74–0.95)	None	None	None
		2) Context “ the qualityof the organizational contextor environment in whichthe research is implemented”	6 items (α = 0.85)								
		3) Facilitation “processesby which implementationis facilitated”	9 items (α = 0.95)								
4- Theorganizationalchangequestionnaire[26]	Bouckenoogheet al (2009)	1) Climate of change orinternal context of change“involves trust in leadership,cohesion and politicking”	5 items	10 Expert judges	Concurrent	ConvergentEFACFAShared group variance known group	Yes	Cronbach alpha (0.69–0.89)	None	None	None
		2) Process of change“how change is dealt with”	3 items								
		3) Readiness-for-change“multifaceted attitudetoward change”	3 items								
5- OrganizationalInformationTechnologyInnovationReadinessScale (OITIRS)[35]	Snyder-Halpern(1996)	1) Resources “IT innovationsupport mechanisms”	6 items(α = 0.83)	None	Concurrent	ConvergentEFACFA	None	Cronbach alpha (0.83–0.92)	None	None	None
		2) End-users “Usercharacteristics and profile”	6 items(α = 0.83)								
		3) Technology “ITinfrastructure”	6 items(α = 0.83)								
		4) Knowledge “Historicalknowledge of external andinternal forces driving pastand current IT innovation decisions”	6 items (α = 0.78)								
		5) Processes “Operationaland work processes thatinfluence IT innovation”	6 items(α = 0.85)								
		6) Values and goals“Individual andorganizational ITvalues and goals”	6 items (α = 0.89)								
		7) Management structures“Organizational andoperational structures thatinfluence IT innovation”	6 items (α = 0.85)								
		8) Administrative support“Leadership style andpractices that influence ITinnovation”	6 items (α = 0.92)								
6- Perceivedorganizationalreadiness forchange (PORC)[36]	Armenakis,Harris andMossholder(1993)	1) Commitment of seniormanagement to the change“how senior management actsduring transformationalchange”	4 items(α = 0.88) (Fornell & Lacker’s = 0.87	None	Predictive	ConvergentEFACFA	None	Cronbach alpha (0.75–0.88)Fornell & Larcker (0.75–0.89)	None	None	None
		2) Competence of changeagents “actions andbehaviours of those whohad been charged withimplementing thechange-change agents”	4 items(α = 0.88)(Fornell & Lacker’s = 0.83)								
		3) Support of immediatemanager “support employeesreceived from theirimmediate managerduring the change process”	3 items(α = 0.75)(Fornell & Lacker’s = 0.75)								
		4) Poor communication ofchange “a list of what can beconsidered the worst practicesin terms of communicatingchange”	3 items(α = 0.88) (Fornell & Lacker’s = 0.89)								
		5) Adverse impact on work“Perception of negative effectsorganizational change onpeople’s work”	5 items(α = 0.75) (Fornell & Lacker’s = 0.76)								
7- ProactiveOrganizationalChange:AssessingCritical SuccessFactors [37]	Nelsonet al (1999)	1) Mission “Internal andexternal stakeholders’perceptions aboutorganizations’ mission”	4 items	None	None	None	None	None	None	None	None
		2) Leadership “promotes andsustains partnerships withinternal and externalstakeholders”	5 items								
		3) Planning “acquisition anddissemination of new ideasfrom outside and inside theorganization”	5 items								
		4) Information “availabilityinformation about communityneeds and resources, aboutclients of the agency, sharinginformation with communitystakeholders”	6 items								
		5) Teamwork “activecollaborationwith communitypartners”	5 items								
		6) Operations “cycletimes to develop newprograms”	4 items								
8- OrganizationalTelehealthreadinessassessment tool[38]	Jennettet al (2004)	1) Organizational corereadiness “addressed theoverall planning process for aproposed e-health program,and the knowledge andexperience of planners withprograms using ICT”	2 items	3 Expert judges	Concurrent	None	Yes	None	None	None	None
		2) Organizational engagementand planning readiness“active participation ofpeople in the idea oftelehealth”	15 items								
		3) Organizational workplacereadiness	6 items								
		4) Organizational technicalreadiness	5 items								
9- e-HealthReadinessmeasure [39]	Poissant,Touré &Swaine (2007)	1) Individual subscale	11 items	Yes	None	None	None	Cronbach alpha (0.85–0.90)	None	None	None
		2) Organizationalenvironment									
		3) Technology									
10- OrganizationCulture andReadinessSurvey(OCRS) [40]	Melnyket al (2008)	1) Extent to which culturalfactors that influencesystem-wide implementationof EBP exist in theenvironment.	19 items	None	None	None	None	Cronbach alpha(≥0.90)	None	None	None
		2) Perceived readinessfor integration of EBP									
11- TeamClimateInventory(TCI) [27]	Anderson&West (1994)	1) Participative safety	38 items	Expert judges	Yes	CFAEFA	Yes	Cronbach alpha (0.84–0.94)	None	None	None
		2) Support for innovation									
		3) Vision									
		4) Task orientation									
12- SociotechnicalSystemsAssessmentSurvey(STSAS) [41]	Pasmore(1988)	1) The Innovativenesssubscale “measures rewardsfor innovation, propensity forrisk taking, and extent towhich the organizationleaders and membersmaintain a futuristicorientation”	10 items	None	None	CFA	None	Cronbach alpha (0.89)	None	None	None
		2) Cooperation subscale“which measure teamwork,flexibility, changes inorganizational structure, andextent to which individualsand subunits work together”	7 items								
13- ComputerizedPhysicianOrder Entry(CPOE)readinessassessmenttool [42]	Stableinet al (2001)	1) External Environment“External and internalforces that are pushing theorganization to implementCPOE”	2 items	None	None	None	None	None	None	None	None
		2) OrganizationalLeadership“The organizations’commitment to CPOEas a top strategic priority”	3 items								
		3) Organizational Structureand Function “Presence andeffectiveness oforganizational structures,relationships, and processesneeded to implement andmaintain CPOE”	3 items								
		4) Organizational Culture“Organizations’ capacity toengage in and sustain largescale change”	3 items								
		5) Care Standardization“Organizations’ ability toadopt or develop standardcare processes and implementthem across the organization”	3 items								
		6) Order ManagementProcess “Present stateof order managementservices”	3 items								
		7) Access to InformationClinician “experience withclinical computing as anelement of routine clinicalwork”	3 items								
		8) Information TechnologyComposition “roles, skills,structure and methodologiesof the IT department”	2 items								
		9) Information TechnologyInfrastructure “Physicalinfrastructure and technicalcomponents of CPOE”	3 items								
14- Assessment ofthe saferpatientsinitiatives(SPI) [43]	Burnettet al. (2010)	1) Culture and attitudestowards quality and safety	15 items	None	None	None	None	None	None	None	None
		2) Systems and infrastructure									
		3) Availability of resources									
15- Notspecified*(Demiris et al)* [44]	Demiriset al. (2007)	1) Motivation of programleaders and staff“motivational forcesinfluencing the need forchange”	27 items	None	None	None	Yes	None	None	None	None
		2) Institutional resources“adequacy of resourcesneeded for daily activitiesand for supporting change”									
		3) Organizational climate“an environment thatencourages adoption ofpractices to make changessustainable”									
16- Not specified(*Hamilton et al)* [45]	Hamiltonet al. (2010)	1) Motivation for change“motivational forcesinfluencing the need forchange”	3 items	None	None	None	None	None	None	None	None
		2) Staff attributes“efficacy and adaptabilityof staff and leaders”	2 items								
		3) Organizational climate“an environment thatencourages adoption ofpractices to make changessustainable”	5 items								
17- Psychometricallysound surveyinstrument [28]	Holt, Armenakis,Feild & Harris(2007)	1) Appropriateness“belief that a change wasnecessary”	10 items	88 Expert judges	Predictive	ConvergentEFACFA	None	None	None	None	None
		2) Management Support“belief that the organizationalleaders were committed to thechange”	6 items								
		3) Change efficacy“belief that the changecould be implemented”	6 items								
		4) Personal valence“belief that the changewould be personallybeneficial”	3 items								
18- Not specified*(Kristensen & Nohr)* [34]	Kristensen,&Nohr (2000)	1) Respondents Knowledge“understanding of theorganisations importancein the entire healthcaresystem and hospital visionsand goals”	52 items	None	None	CFA	None	None	None	None	None
		2) Respondents’ view of needin the organization of thechanges “make the visionsand goals of the hospitalattainable”									
		3) Respondent’s opinion ofchange in the organization, inthe specific IT context									
		4) Respondent’s opinion ofthe organization’s ability toplan and carry out the changes									
19- GeriatricInstitutionalAssessmentProfile (GIAP)[46],	Boltzet al. (2002)	1- Geriatric NursingKnowledge/Attitudes Scale“Nurses’ knowledge andattitude toward restraint use,sleep, incontinence andpressure ulcers”	22 items	Yes	None	EFACFA	Yes	Cronbach alpha (0.90)	None	None	Yes
		2- Geriatric Care Environment(GCE) Scale “hospital staffperceptions of the intrinsicand extrinsic factors thatshape the geriatricinstitutional milieu”(subscales: Institutionalvalues regarding older adultsand staff; Capacity forcollaboration; Resourceavailability; Aging-sensitivecare delivery)	28 items								
		3-Professional issue(subscales: Staff disagreement,Staff/family/patientdisagreement, Geriatricresource use, Perceived legalvulnerability, Perceivedupsetting behaviours, Burdenof upsetting behaviours)	47 items								
20- Long-Termcare (LTC)readiness tool[48]	Cherry(2011)	1) Organizationalculture/human factors“refers to leadership support,employee attitude andcongruence withorganizational mission”	4 items	Yes	None	None	None	Cronbach alpha (0.95)	None	None	None
		2) Implementationprocesses/staff training“refers to specific needs,implementation planning andtraining”	9 items								
		3) Technical requirements“refers to technical supportand physical plantrequirements”	3 items								
21- Notspecified(*Bobiak el al)* [49]	Bobiaket al. (2009)	1) Practice members’motivations “Motivation(intentions or desires)represented by statements orexhibited behaviors to makean effort toward a new orexisting goal”	25 items	None	None	Convergent EFA	None	Cronbach alpha(0.94)	None	None	None
		2) Resources for change“Tangible practice assets orintangible strengths, attitudes,and skills of its members thatmay enable change”									
		3) Perceived options forchange “The extent to whichpractice members understand,evaluate, or reflect onopportunities for change”									
		4) External influences“External organizations,events, or contextual featuresthat can affect or be affectedby the practice”									

EFA, Exploratory factor analysis; CFA, Confirmatory factor analysis.

### Quality of reporting of psychometrical characteristics

Consistent with the literature on psychometric properties in the AERA/APA/NCME standards, we proposed our own system for evaluating the reporting of psychometrical properties in ORC measurement instruments. The checklist included seven items scored yes (1) or no (0), addressing four advanced sources of validity evidence (i.e., content, response processes, internal structure, relations to other variables) and three categories of reliability evidence (i.e., internal consistency-Cronbach’s alpha, parallel forms coefficients, test-retest reliability) [22].

Validity, according to the SEPT, refers to the extent to which a measure achieves the purpose for which it is intended and is determined by the “degree to which evidence and theory support the interpretations of test scores entailed by proposed uses of tests…” ([22], p.9). As outlined in the SEPT [22], validity is a unitary concept with all validity evidence contributing to construct validity. Content evidence refers to the extent to which the items in a self-report measure adequately represent the content domain of the concept or construct of interest. Experts’ evaluations are key approaches for obtaining content validity evidence. Response processes evidence refers to how respondents interpret, process, and elaborate upon item content and whether this behavior is in accordance with the concept or construct being measured. Internal structure evidence refers to the degree to which individual items fit the underlying construct of interest. Factor analysis (exploratory and confirmatory) or internal consistency reliability are commonly used to provide internal structure validity evidence. Evidence on relations to other variables provides the fourth source of validity evidence. It is an umbrella term that refers to test-criterion relationships demonstrated through concurrent and predictive validity methods and to evidence base on convergent and discriminant relations, which where historically subsumed in the construct validity [22].

According to the SEPT [22], instrument reliability is defined as “the consistency of measurements when the testing procedure is repeated” ([22], p.25). Reliability may be estimated in terms of one or more reliability coefficients, depending on which approach is used for replicating the instrument. Three categories of reliability coefficients are reported: internal consistency-Cronbach’s alpha, test-retest reliability and parallel forms coefficients [22]. For instance, Cronbach’s alpha values greater than 0.8 are considered strong indicators of reliability [24].

In addition to summarizing the psychometric properties of the 26 identified measurement instruments, we assessed the overall instrument reliability and validity with a score ranging from 0 to 4 for the validity evidence and from 0 to 3 for the reliability evidence. We gave a score of “1” for each of the standards complied and a score of “0” if the standard was not addressed or not achieved (Table 4). An overall instrument rating is also included in Table 4.

**Table 4:** Assessment checklist for psychometric properties according to AERA/APA/NCME standards for Educational and Psychological Testing.

Instrument’ name	Validity eviden					Validity instrument score/4	Reliability			Reliability instrument score/3	Overall Instrument rating/7
	Content	Response processes	Internal structure	Relations to other variables			Internal consistency	Parallel forms	Test-retest		
				Convergent/discriminant	Test-Criterion						
1-a Organizational readiness for change scale (ORC) [25]	X	X	√	X	√	2	√	X	X	1	3
1-b Extended organizational readiness for change scale (ORC) [21], [30]	X	X	√	√	√	2	√	X	X	1	3
1-c Modified ORC scale [50]	X	X	√	X	X	1	√	X	X	1	2
1-d TCU-ORC [25]	√	√	√	√	X	4	√	X	X	1	5
1-e Modified Texas Christian University – Director version (TCU-ORC-D) [29]	√	X	√	X	X	2	√	X	√	2	4
1-f Functional Organizational Readiness For Change Evaluation (FORCE) [51]	X	X	√	X	X	1	√	X	X	1	2
2- The Medical Organizational Readiness For Change (MORC) [52]	X	X	√	X	X	1	√	X	X	1	2
3- Organizational readiness to change assessment instrument (ORCA) [53]	X	X	√	√	X	2	√	X	X	1	3
4- The organizational change questionnaire [26]	√	X	√	√	√	3	√	X	X	1	4
5- Organizational Information Technology Innovation Readiness Scale (OITIRS) [35]	X	X	√	√	√	2	√	X	X	1	3
6- Perceived organizational readiness for change (PORC) [36]	X	X	√	√	√	2	√	X	X	1	3
7- Proactive Organizational Change: Assessing Critical Success Factors [37]	X	X	X	X	X	0	X	X	X	0	0
8- Organizational Telehealth readiness assessment tool [38]	√	X	X	X	√	2	X	X	X	0	2
9- e-Health Readiness measure [39]	√	X	√	X	X	2	√	X	X	1	3
10- Organization Culture and Readiness Survey (OCRS) [40]	X	X	√	X	X	1	√	X	X	1	2
11- Team Climate Inventory (TCI) [27]	√	X	√	X	√	3	√	X	X	1	4
12- Sociotechnical Systems Assessment Survey (STSAS) [41]	X	X	√	X	X	1	√	X	X	1	2
13- Computerized Physician Order Entry (CPOE) readiness assessment tool [42]	X	X	X	X	X	0	X	X	X	0	0
14- Assessment of the safer patients initiatives (SPI) [43]	X	X	X	X	X	0	X	X	X	0	0
15- Not specified *(Demiris et al)* [44]	X	X	X	X	X	0	X	X	X	0	0
16- Not specified (*Hamilton et al)* [45]	X	X	X	X	X	0	X	X	X	0	0
17- Psychometrically sound survey instrument [28]	√	X	√	√	√	3	X	X	X	0	3
18- Not specified *(Kristensen & Nohr)* [34]	X	X	√	X	X	1	X	X	X	0	1
19- Geriatric Institutional Assessment Profile (GIAP) [46], [47]	√	X	√	X	X	2	X	X	√	1	3
20- Long-Term care (LTC) readiness tool [48]	√	X	√	X	X	2	√	X	X	1	3
21- Not specified (*Bobiak el al)* [49]	X	X	√	√	X	2	√	X	X	1	3

## Results

### Flow of studies

The initial search strategy identified 3711 references after duplicates were removed. After screening using the inclusion criteria, we retained 39 publications describing 26 ORC measures relevant for health care organizations (Figure S1). One hundred eight studies were excluded since they did not refer specifically to OR, did not concern the health care domain, or were limited to individual-level measure of readiness.

### Characteristics of ORC measurement instruments

Of the 26 instruments measuring ORC retained, some were adaptions of existing scales. For instance, we found six versions of the Organizational Readiness for Change scale (ORC) that were developed by adding or modifying constructs from the original version created by Lehman et al. [25]. Of the 26 ORC measurement instruments, 16 (62%) were developed following an underlying conceptual purpose or theoretical foundation. For the 10 (38%) remaining instruments, authors did not refer to an underling theory or conceptual framework. Five (19%) of the 26 measurement instruments were developed before 2000, nine (35%) between 2000 and 2005, and 12 (46%) after 2005. Half of the included studies (50%) presented methodological development and/or psychometric validation of the instrument, and the other 50% were empirical assessment of the tools (e.g., applicability of the instrument in a specific context) (Table 2).

### Psychometric assessment of instruments

We reviewed the psychometric standards regarding validity and reliability, as reported by the authors of the papers presenting the 26 identified instruments, based on the AERA/APA/NCME Standards for Educational and Psychological Testing - SEPT [22] (Table 4). According to the SEPT, 18 (69%) measurement instruments complied with both validity and reliability criteria, based on the information reported by authors of the retained articles. Twenty one (21) instruments reported at least one of the four validity criteria. In most of the studies, authors did not report whether they assessed all sources of validity or reliability evidence. We found that evidence for internal structure was reported for twenty (77%) instruments through performing statistical analysis (e.g., factor analysis, internal consistency reliability). Response processes validity evidence was reported for only one (4%) instrument, namely the *Texas Christian University-ORC (TCU-ORC) scale*. Authors outlined relations to other variables based on predictive and/or concurrent, convergent and/or discriminant validity evidence for eight (31%) ORC measurement instruments. Content validity, as determined by a review of expert judges, was reported for nine (35%) of the 26 instruments. The highest instrument validity score (4 out of 4) was obtained for the *Texas Christian University-ORC (TCU-ORC) scale*, meaning that authors of the papers reporting this instrument provided all four sources of validity evidence.

Authors of retained papers outlined estimates of reliability for 18 (69%) of the 26 identified measurement instruments. The most common form of reliability testing used for these 18 instruments was internal consistency. This form of reliability testing was found to be present in 17 of the papers. No information was provided by authors on parallel forms reliability for any of the 26 instruments. The most widely used coefficient was the Cronbach’s alpha. Papers reported test-retest reliability for two (8%) instruments, namely the *Geriatric Institutional Assessment Profile (GIAP)* and the *Modified Texas Christian University – Director version (TCU-ORC-D)*. Papers related to the *Modified Texas Christian University – Director version (TCU-ORC-D)* provided the highest reliability score (2 out of 3).

Finally, no information was provided by authors of retained articles regarding the reliability and validity of five (19%) of the 26 identified instruments (Table 4).

## Discussion

This systematic review aimed to assess the current literature regarding the psychometric properties of instruments developed to measure ORC in the health care context at the organizational level. We identified 26 instruments – described in 39 publications – for measuring ORC that were relevant for health care organizations. This leads us to two main observations.

First, overall, we found limited evidence of reliability or validity reported for the 26 identified instruments measuring ORC in the health care domain at the organizational level. Eighteen (69%) measurement instruments partly complied with both validity and reliability standards. For instance, evidence of assessing the four sources of validity – content, response processes, internal structure and relations to other variables consistent with the construct validity – and the internal consistency reliability was reported for only one instrument, the TCU-ORC scale [25] However, no information was reported for test-criterion relationships for the TCU-ORC scale. Of the 26 identified ORC measurement instruments, three additional instruments, namely the Organizational change questionnaire [26], the Team Climate Inventory (TCI) [27] and the Psychometrically sound survey instrument [28] have undergone an assessment of reliability, and of three sources of validity evidence in terms of content, internal structure and relations to other variables. According to the checklist that we developed based on the SEPT, the only instrument for which authors reported all validity standards (4 out of 4) was the *TCU-ORC scale* [25]. The highest score for reporting reliability standards (2 out of 3) was attributed to the *Modified Texas Christian University – Director version (TCU-ORC-D)* [29].

Second, we believe that the 18 new instruments measuring ORC in healthcare organizations identified in this systematic review update and complement the work of Weiner’s et al. [8] and Holt’s et al. [9], but our review is distinct because it focuses on available valid and reliable measurement instruments that could be applied to KT in the health-care sector, at the organizational level in particular. To do so, we developed a systematic checklist to evaluate the quality of reporting, based on the AERA/APA/NCME Standards for Educational and Psychological Testing-SEPT. In Weiner et al.’s review, only eight instruments assessing readiness in healthcare organizations – on a total of 43– were reported. Only three instruments assessing readiness at the healthcare organizational level had undergone systematic assessment of validity and reliability. Supporting the findings by Weiner et al. [8], Holt et al. [9] also reported the limited evidence of reliability and validity of most currently available instruments in health care and other contexts. By reviewing the literature on ORC measurement instruments in private and public sector organizations, Holt et al. [9] systematically classified and described 32 different instruments assessing organizational readiness. Only two instruments showed evidence of content, construct, and predictive validity. The use of scales with limited prior assessment of reliability or validity is a concern [30]. According to Kimberlin and Winterstein [31], validity requires that an instrument is reliable, but an instrument can be reliable without being valid. Reliability is a necessary, but not sufficient, component of validity [32]. An instrument that does not yield reliable scores does not permit valid interpretations [33]. Evidence should be sought from several different sources to support any given interpretation, and strong evidence from one source does not obviate the need to seek evidence from other sources [33]. Ideally, key indicators of the quality of a measuring instrument are the reliability and validity of the constructs [31]. These findings should be considered preliminary and suggestive of the need for further refinement in ORC measurement. Additional psychometric testing of instruments designed to measure ORC is needed. Weiner et al. [8] concluded that researchers need to give greater attention to measurement development, testing, and refining.

Five years after Weiner et al.' s review, our findings indicate little improvement in the development of ORC measurement instruments. A lack of instruments specifically designed to assess organizational readiness for knowledge translation in health care or existing instruments that could be used for this purpose was observed. We identified a limited number of valid and reliable measurement tools that could be readily used in health care settings to assess the degree of readiness to implement evidence-based change. The findings of our review lay groundwork for the development of a comprehensive instrument based upon frameworks identified in a previous work [21] to assess OR for KT needed to support implementation of evidence-based practices.

### Limitations

Although this review updates current knowledge on available ORC instruments, it has some limitations. First, we used narrow inclusion criteria in order to focus on ORC instruments that were developed or applied in the field of health care services. However, ORC measurement instruments developed in other fields could potentially be relevant to health care. Second, we did not contact the authors of the identified measurement instruments to validate our analysis or ask them more information about their tools. Thus, our evaluation of the compliance of the measurement tools with the SEPT is based on what is reported in the articles and a negative score does not necessary means that the assessment of validity and reliability has not been done, but rather that the authors did not report it in their publication.

### Conclusion

Overall, our review identified 26 instruments for measuring ORC in the health care context described in 39 publications. Our findings indicate little improvement in the development of ORC measurement instruments that could be applied to KT in the health care sector. We found limited evidence of reliability or validity for the 26 identified instruments measuring ORC in the health care domain at the organizational level. Only 18 (69%) of the 26 measurement instruments complied with both validity and reliability criteria proposed by the AERA/APA/NCME Standards for Educational and Psychological Testing. The TCU-ORC instrument got a score of 4 out of 4 for validity testing, and 2 out of 3 for reliability testing. This instrument could thus provide a good basis for assessing organizational readiness for knowledge translation in health care.

## Supporting Information

Figure S1 **Study selection flow diagram.**
(PDF)

Checklist S1 **PRISMA checklist.**
(PDF)
