# Supplementary material for: A Systematic Review of Instruments to Assess Organizational Readiness for Knowledge Translation in Health Care
Source: PLoS One. 2014 Dec 4;9(12):e114338. doi: 10.1371/journal.pone.0114338 (PMC4256226; doi:10.1371/journal.pone.0114338)
Supplement: Figure S1 — Study selection flow diagram. (PDF) [file pone.0114338.s001.pdf]

PRISMA 2009 Flow Diagram

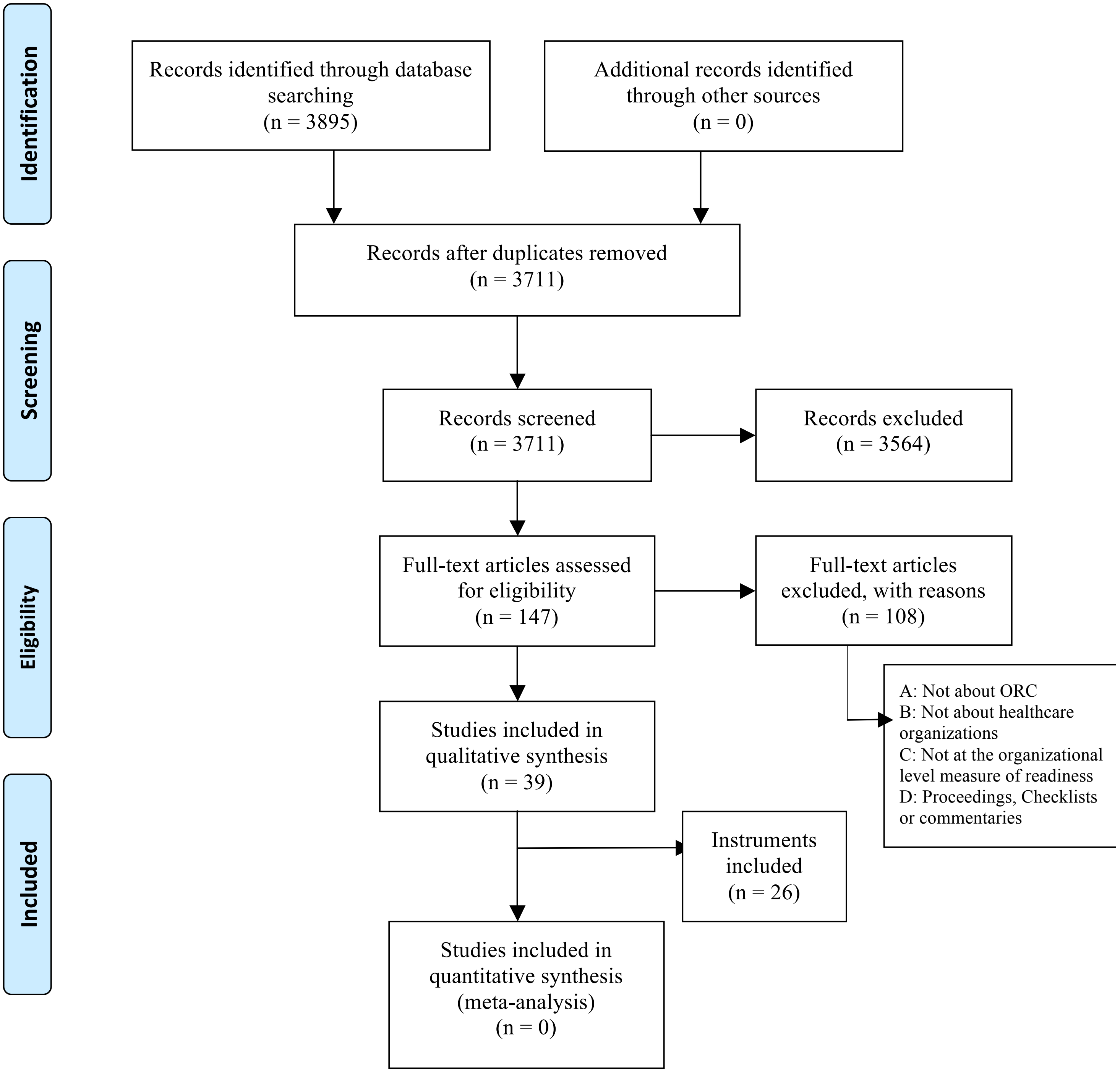

Figure 1

From: Moher D, Liberati A, Tetzlaff J, Altman DG, The PRISMA Group (2009). *P*referred *R*eporting *I*tems for *S*ystematic Reviews and *M*eta-*A*nalyses: The PRISMA Statement. PLoS Med 6(6): e1000097. doi:10.1371/journal.pmed1000097

For more information, visit www.prisma-statement.org.
